# A novel bacteriocin from *Enterococcus faecalis* 478 exhibits a potent activity against vancomycin-resistant enterococci

**DOI:** 10.1371/journal.pone.0186415

**Published:** 2017-10-12

**Authors:** Uraporn Phumisantiphong, Kanokrat Siripanichgon, Onrapak Reamtong, Pornphan Diraphat

**Affiliations:** 1 Department of Microbiology, Faculty of Public Health, Mahidol University, Bangkok, Thailand; 2 Department of Molecular Tropical Medicine and Genetics, Faculty of Tropical Medicine, Mahidol University, Bangkok, Thailand; Universidad Nacional de la Plata, ARGENTINA

## Abstract

The emergence of multidrug-resistant enterococci (MDRE) and particularly vancomycin-resistant enterococci (VRE) is considered a serious health problem worldwide, causing the need for new antimicrobials. The aim of this study was to discover and characterize bacteriocin against clinical isolates of MDRE and VRE. Over 10,000 bacterial isolates from water, environment and clinical samples were screened. *E*. *faecalis* strain 478 isolated from human feces produced the highest antibacterial activity against several MDRE and VRE strains. The optimum condition for bacteriocin production was cultivation in MRS broth at 37°C, pH 5–6 for 16 hours. The bacteriocin-like substance produced from *E*. *faecalis* strain EF478 was stable at 60°C for at least 1 hour and retained its antimicrobial activity after storage at -20°C for 1 year, at 4°C for 6 months, and at 25°C for 2 months. A nano-HPLC electrospray ionization multi-stage tandem mass spectrometry (nLC-ESI-MS/MS) analysis showed that the amino acid sequences of the bacteriocin-like substance was similar to serine protease of *E*. *faecalis*, gi|488296663 (NCBI database), which has never been reported as a bacteriocin. This study reported a novel bacteriocin with high antibacterial activity against VRE and MDRE.

## Introduction

Vancomycin-resistant enterococci (VRE) and multidrug-resistant enterococci (MDRE) have emerged as hospital-acquired pathogen over the last three decades [1]. Among various enterococci, *Enterococcus faecalis* and *Enterococcus faecium* are known to be the most common causes of nosocomial infections, such as urinary tract infection, surgical wound infection, pneumonia, endocarditis, bacteremia and meningitis [2]. They are intrinsically resistant to many antimicrobial agents used in clinical setting and rapidly acquire resistance genes and mutations [1, 3]. Enterococci can grow in a wide range of pHs and temperatures, high salt concentration and persist on non-living objects for weeks, allowing them to survive in hospitals and extreme environments [4, 5]. Generally, vancomycin is reserved for certain serious infections, which are non-responsive to other antibiotic treatment. Since the first VRE case report in Europe in 1980, the prevalence of VRE has increased dramatically in various countries [6, 7]. VRE can cause serious infections, especially endocarditis and bacteremia, in immunocompromised patients or patients on treatment with antibiotics such as cephalosporins [8]. The search for new effective antimicrobial agents has become a global concern due to the increasing prevalence of MDR-and VRE. In the past three decades, the process of new antibiotic discovery has slowed and only few antibiotics against VRE have been developed [9]. Furthermore, some enterococci have evolved resistance to the most recent antimicrobial agents such as quinupristin-dalfopristin, linezolid, daptomycin and tigecycline during therapy [3]. The number of antibiotics resistant enterococci has been continually rising while the number of effective antibiotics has been declining.

Antimicrobial peptides (AMPs) have drawn attention as alternatives to conventional antibiotics. Bacteriocins are bacterial antimicrobial peptides that have been used in the food industry for over 50 years and are generally recognized as safe (GRAS) [10]. They are also used to treat bacterial infections and control antibiotic-resistant bacteria, either used alone or in combination with conventional antibiotics [11–13]. Bacteriocins kill target bacteria by several mechanisms, including pore formation in the cell membrane, inhibition of cell wall- or protein synthesis, and degradation of cellular DNA [14]. The rise in interest toward bacteriocins is due to their potent inhibitory activities, the fact that they are safe for humans, their stability and various modes of action. Furthermore, resistance toward bacteriocins has been rarely observed [15, 16]. Bacteriocin producing bacteria have been isolated from plants, animals, food, water and soil [17–21].

Although bacteriocins have been extensively studied, it was generally in regard to their application in the food industry as preservative. In addition, there is a scarcity of reports about the efficacy of bacteriocins against vancomycin-resistant and multidrug-resistant enterococci clinical isolates. Therefore, the aims of this study were to discover and characterize bacteriocins active against clinical isolates of antimicrobial resistant *E*. *faecium* and *E*. *faecalis*.

## Materials and methods

### Sample collection and bacterial isolation

Bacterial isolates were collected from environmental water, laboratory environment and clinical samples during July 2014 and April 2015 for bacteriocin-production screening. The water samples were collected from Mahasawat Canal, Bangtal Canal, Chao Phraya River Bangkok and Pasak River Saraburi, (S1 Table). The sample sources are communal water supplies, no specific permissions were required for the samples collection. The environmental and clinical samples were collected from the Faculty of Medicine Vajira Hospital, Navamindradhiraj University, Bangkok, Thailand with the approval by the institute’s Ethical Review Committee for Human Research.

The water samples were pre-filtrated using 0.8 μm cellulose nitrate membranes (Nalgene Company, Rochester, USA) to remove large particles and then passed through 0.2 μm Supor membranes to concentrate bacterial cells (Pall Corp, USA). The membranes were then cut and immersed in Luria-Bertani broth (LB) (Merck, KGaA, Darmstadt, Germany) supplemented with 10% fetal bovine serum and incubated with shaking at 37°C for 4 hours (200 rpm, Innova 4300 incubator shaker, New Brunswick Scientific, Edison, NJ). The cultures were then isolated on LB agar.

Environmental samples were collected from laboratory-bench-surfaces (10 cm^2^) in the hospital using sterile moisten cotton swabs. Each sample was inoculated onto blood and MacConkey agar plates, and incubated at 37°C overnight. Bacterial colonies of all morphology were screened for bacteriocin production. The isolates from stool and rectal swab cultures with Enterococcus-like morphology on blood agar were identified and screened for bacteriocin production (S1 Fig).

*Enterococcus faecalis* ATCC 51299 and *E*. *faecium* ATCC 35677, as well as clinical isolates of MDR-enterococci were used as indicators for antimicrobial activity.

### Screening for *E*. *faecalis* and *E*. *faecium* inhibitors

The inhibitory activity against *E*. *faecalis* and *E*. *faecium* was preliminary detected using the bacterial inhibition test. Briefly, the indicators were cultured in LB broth overnight at 37°C. Cell density was then adjusted to ~ 10^7^ cells/ml using sterile normal saline solution and swabbed onto the surface of LB agar plates. The tested bacterial isolates were cross streaked onto the lawn of the indicators the plates incubated at 37°C overnight. The zone of inhibition was observed and measured.

### Screening for bacteriocin production

The tested isolates that produced large inhibition zone against both indicators were selected to screen for the production of bacteriocin by spot-on-lawn method [22]. Briefly, cell free supernatants (CFSs) of the tested strains were prepared by growing one colony of each tested strains in 10 ml of LB broth at 37°C for 16–18 hours. The cultures were centrifuged at 11,000 x *g*, 4 °C for 10 minutes (IEC Multi RF, Thermo Electron Corporation, USA) and the supernatant were sterilized by passing through a 0.2 μm pore size filters (Acrodisc® with Supor membrane, Pall Corp., USA). The indicator lawns were prepared by adding each indicator to 3 ml of molten LB medium at the final concentration of 10^7^ cells/ml and overlaid on Muller-Hinton agar plate. After solidification, 10 μl of each CFSs was spotted onto the indicator lawn, incubated at 37°C overnight and the clear inhibition zone were observed.

### Phage plaque assay

In order to confirm that the inhibitory activity was not due to bacteriophages, the phage plaque assay was performed with some modification [23]. In brief, overnight cultures of tested isolates were transferred (1:100) to fresh LB broth supplemented with mitomycin C (Ametycin^®^ and TCI, Japan) to a final concentration of 1 μg/ml and incubated at 37°C for 4 hours. The bacterial cells were removed by centrifugation at 11,000 x *g* for 10 minutes and the supernatants sterilized through a 0.2 μm filter membranes.

The indicators were cultured overnight and diluted with normal saline solution to a final concentration of approximately 10^8^ CFU/ml. One hundred microliters of the indicator strains were either mixed with 10 μl of a mitomycin C-induced CFSs or 10 μl of a mitomycin C (control), and incubated at 37°C for 30 minutes. The treated cells were added into 3 ml of molten LB medium and overlaid on LB plate. The plates were incubated overnight at 37°C and examined for plaque formation.

### Bacteriocin production confirmation

Besides bacteriocins, some bacteria can produce organic acids, hydrogen peroxide (H_2_O_2_) and other antimicrobial substances that inhibit growth of sensitive bacteria. To eliminate the possibility of the interference from organic acids and H_2_O_2_, the CFSs were adjusted to pH 7 and treated with catalase enzyme (1 mg/ml, Sigma Chemical Co., Dorset, UK) for 1 hour at 37°C before antimicrobial activity testing. The CFSs were tested against *E*. *faecalis* and *E*. *faecium* using the agar well diffusion method [24].

The indicator plates were prepared as described in the spot-on-lawn method. After drying, 6 mm wells were bored and filled with 50 μl of two fold serial dilution of the CFSs. Sterile MRS broth was used as negative control. The activity was expressed in arbitrary unit (AU/ml), the reciprocal of the highest dilution that inhibit the growth of the indicator strains [25]. The assay done in triplicate for at least two independent experiments and the average value was reported.

### Inhibitory spectrum of EF478 against MDRE and VRE

To determine the inhibitory spectrum of bacteriocin EF478, the antimicrobial activity of this bacteriocin was evaluated against 68 clinical isolates of MDRE and VRE using the spot-on-lawn method described above.

### Identification of bacteriocin-producing strain

The producing strain that showed the strongest inhibitory activity against *E*. *faecalis* and *E*. *faecium* was identified by conventional method and the automated MicroScan, Walk-Away system (Siemens Healthcare Diagnostics, USA) using 96 well gram-positive combination panels (PC21). Results were interpreted using the Lab Pro program, version 3.01, following the Clinical and laboratory Standards Institute guidelines [26]. The strain was further characterized by MALDI-TOF-MS (autoflex™ speed MALDI-TOF/TOF, Bruker Daltonics, Germany) using flexControl version 3.4. The obtained results were analyzed by comparing the raw spectra with the spectra of the company’s library and expressed as score. The score ranged from 0 to 3 as recommended by the manufacturer. Score values of >1.7 generally indicated relationships at the genus level, and values of >2.0 generally indicated relationships at the species level. The highest score was used for species identification.

### Bacteriocin production optimization

The optimal condition for bacteriocin EF478 production was evaluated by varying culture media, medium pH, and incubation temperature and culture length. Three growth media namely Luria-Bertani medium (LB), De Man Rogosa Sharpe medium (MRS) and brain heart infusion broth (BHI) (all were purchased from Merck, Darmstadt, Germany) were used for comparison. After incubation at 37°C overnight, the antimicrobial activity of the CFS derived from each growing medium was tested against *E*. *faecalis* ATCC 51299 by agar well diffusion method. The medium with the highest antimicrobial activity was selected for further tests.

Optimal pH for the production of bacteriocin EF478 was evaluated by adjusting culture medium from pH 4 to pH 8 using 1 N HCL or 1 N NaOH. After inoculation and incubation at 37°C overnight, the culture media were readjusted to pH 7 before being measured for its antimicrobial activity against *E*. *faecalis* ATCC 51299.

To establish the optimal temperature for bacteriocin production, EF478 was inoculated in the selected medium and pH and incubated with overnight shaking at 25°C, 37°C and 42°C. The CFS derived from each incubation temperature was examined for the antimicrobial activity. To study the kinetics of bacteriocin production, the EF478 was cultivated in the optimal medium, pH and temperature. Aliquots of the sample were analyzed for the antibacterial activity at 0, 8, 16, 24, 32, 40, 48 and 56 hours after incubation.

Mitomycin C, a DNA damaging agent, has been used to induce several bacteriocin production. Mitomycin C (Ametycin^®^, TCI, Japan) was added to the mid-log phase culture of the EF478 at the final concentration of 0.25 mg/ml, 0.50 mg/ml and 1 mg/ml. After 4 hours incubation, the CFSs were prepared and protein concentration determined by Bradford methods (Bio-Rad Laboratories Inc., Hercules, CA, USA) [27]. Antimicrobial activity against *E*. *faecalis* ATCC 51299 was determined by agar well diffusion method using non-induced supernatants as a control. All of the assays were done in triplicate for at least two independent experiments and the average values were reported.

### Susceptibility to enzymes, heat and pH treatment

The susceptibility of bacteriocin EF478 to proteolytic cleavage, enzymatic activities, heat treatments and different pH levels was examined. The effect of 1 mg/ml of proteinase K, trypsin, chymotrypsin, amylase and lipase (Sigma Chemical Co., Dorset, UK) on the bacteriocin activity was determined after co-incubation at 37°C for 2 hours. The residual antibacterial activity of the enzyme-treated bacteriocin was then measured against *E*. *faecalis* ATCC 51299 by agar well diffusion method. CFS without enzyme treatment was used for comparison.

The heat stability of the bacteriocin EF478 was evaluated by exposing the bacteriocin to various temperatures (37°C, 60°C, 80°C, 100°C) for 30 min or autoclaved at 121°C for 15 min. The remaining antimicrobial activity against *E*. *faecalis* ATCC 51299 of the cooled samples was tested by agar well diffusion method.

The effect of pH on the bacteriocin was assessed by adjusting the pH of the crude bacteriocin EF478 from 2 to 12 using either 1 N hydrochloric acid (HCl) or 1 N sodium hydroxide (NaOH). After storage at 25°C for 3 hours, samples were readjusted to pH 7 and the residual antimicrobial activity determined by agar well-diffusion method. All of the assays were done in triplicate for at least two independent experiments and the average values were reported.

### Storage condition

Stability of the bacteriocin EF478 after storage at various temperature was determined. The CFS aliquot of 1 ml aliquot tubes at three different temperatures (25°C, 4°C, and -20°C). The antimicrobial activities of stored aliquots and fresh CFS were compared by agar well-diffusion method against *E*. *faecalis* ATCC 51299.

### Concentration and purification of bacteriocin

Bacteriocin EF478 was size-fractionated and concentrated using Amicon Ultra 30K, 10K (Amicon, Millipore Co., Germany) and Macrosep 3K (Pall Macrosep®, USA), respectively. The concentrated protein from each fraction was resolved in SDS-PAGE with 12% (v/v) separating gel and antibacterial activity was detected by gel overlay method. The active fraction was further applied to reversed-phase chromatography using a LiChrolut RP-18 column (40–63 μm) (Merck KGaA, Darmstadt, Germany). The protein sample was eluted with a linear gradient (20% to 80%) of Milli-Q water-acetonitrile containing 0.1% formic acid. The antimicrobial activity of each purified fraction was examined against *E*. *faecalis* ATCC 51299.

### Molecular mass and amino acid sequence determination

The protein band with antibacterial activity was cut and subjected to in-gel tryptic digestion. Tryptic peptide sample were analyzed for amino acid sequences using maXis II^TM^ ESI–QTOF (Bruker Daltonics) coupled with an UltiMate 3000 nano-LC system (Dionex, Surrey, UK). The peptide MS fragments were searched against the non-redundant National Center for Biotechnology Information GenBank database (NCBI, www.ncbi.nlm.nih.gov/) and Bacteria (Eubacteria) database using version 2.5.1.2 of the MASCOT search engine (Matrix Science, London, UK, http://www.matrixscience.com). The search parameters were trypsin digest, monoisotopic mass, and allowing a maximum of one missed cleavage. Peptide and fragment mass tolerance were set as 1.2 Da and 0.6 Da, respectively. Variable modifications were set to carbamidomethylation of cysteine and oxidation of methionine. The instrument type was specified as ESI-QUAD-TOF. Proteins matched with scores over 65 were considered to be significant (*p*< 0.05). Proteins were validated base on protein scores, peptides matches, and percent sequence coverage.

### DNA sequence of the bacteriocin gene

PCR amplification and nucleotide sequencing of the gene coding bacteriocin EF478 was carried out. Genomic DNA of *E*. *faecalis* 478 was extracted using NucleoSpin® Tissue kit (Macherey-Nagel, Bethlehem, PA) according to the manufacturer’s recommendations. The primers were designed based on the sequence that best matched the peptide mass fingerprinting. The primers SP-EF478- forward: ATGAAAAAACGCCTGTTTGCGA and SP-EF478-reverse: CGCGCTATGGCCCACAAT were synthesized by Sigma-Aldrich (SigmaAldrich Co., St. Louis, MO, USA). The reaction mixture contained 100 ng of DNA template, 1x Phusion HF Buffer, 200 μM of each dNTPs, 500 nM of each primers, 1 U Phusion High-Fidelity DNA Polymerase (Finnzymes Oy, Vantaa, Finland), and deionized water to a final volume of 50 μl. PCR were performed using the following conditions: an initial denaturation at 98°C for 2 minutes, followed by 35 cycles of denaturation at 98°C for 10 seconds, annealing at 64°C for 30 seconds, extension at 72°C for 30 seconds, and a final extension at 72°C for 5 minutes. Nucleotide sequencing of the PCR product was accomplished by Solgent Co. Ltd. (Daejeon, South Korea). The sequence was compared to the non-redundant nucleotide collection of the National Center for Biotechnology Information (NCBI) databases using standard nucleotide BLAST (BLASTN) (http://www.ncbi.nlm.nih.gov/BLAST). The sequence was also searched for homology with bacteriocin published in databases, including BACTIBASE [28] and BAGEL [29].

### Protein structure and function prediction

The *E*. *faecalis* serine protease WP_002367871 was used to compare with the EF478 bacteriocin. The PDB files and the ribbon structures were created using I-TASSER server [30–33] and CCP4mg molecular graphics software [34], respectively.

### Ethics statement

This study was approved by the Ethical Review Committee for Human Research Faculty of Public Health, Mahidol University and Faculty of Medicine Vajira Hospital, Navamindradhiraj University. The sources of water samples were communal water supplies, no specific permissions were required for water collection at these locations. The field studies did not involve endangered or protected species.

## Results

### Antimicrobial activity of bacterial isolates

From all bacterial isolates, about 1.6% (165/10, 215) inhibited the growth of one or both indicator strains (Table 1) with inhibition zone varying from 2 to 12 mm. The largest inhibition zone (12 mm) was observed from a clinical isolate. Twenty isolates were selected to prepare the CFSs based on their inhibitory activities against both indicator strains.

**Table 1:** Sources of isolated bacteria and number of isolates (%) that showed inhibition of indicator strains.

Sources	No. of isolates	Isolates with growth inhibition (%)			
		Total	Antibacterial activity against		
			*E*. *faecalis* *ATCC51299	*E*. *faecium* *ATCC35667	Both
Water	9,900	81(0.82)	40 (0.40)	38 (0.38)	3 (0.03)
Environment	180	18 (10)	8 (4.44)	7 (3.89)	3 (1.67)
Clinical**	135	66 (48.89)	28 (20.74)	23 (17.04)	15 (11.11)
Total	10,215	165 (1.62)	76 (0.74)	68 (0.67)	21 (0.21)

*ATCC, American Type Culture Collection

**Stool and rectal swab samples

The twenty CFSs were examined for their inhibitory activity using the spot-on-lawn assay. Only, 4 out of 20 CFSs exhibited potent inhibitory activity against the indicator strains. For these 4 strains (EF 344, EF 349, EF 355 and EF478), the CFSs minimal inhibitory concentration against *E*. *faecalis* ATCC 51299 and *E*. *faecium* ATCC 35667 was measured using agar-well diffusion method. EF 355 and EF478 exhibited the highest activity (320 AU/ml) of the 4 isolates, against both indicators (Table 2).

**Table 2:** Antimicrobial activity of four CFSs against indicators *E*. *faecalis* ATCC 51299 and *E*. *faecium* ATCC 35667 as measured by agar well diffusion assay.

Test isolates	CFS activity (AU/ml) against	
	*E*. *faecalis* ATCC51299	*E*. *faecium* ATCC35667
EF344	80	80
**EF355**	**320**	**320**
EF349	160	80
**EF478**	**320**	**320**

AU/ml: arbitrary unit

The antimicrobial activity of both EF 355 and EF478 was then tested against 68 clinical isolates of MDRE and VRE. EF478 was able to inhibit 28 from 68 (41.1%) clinical isolates, whereas EF355 inhibited 26 of 68 isolates (38.2%). Therefore, we selected EF478 for further study and characterization.

### Characterization of the bacteriocin EF478

**Fig 1:**
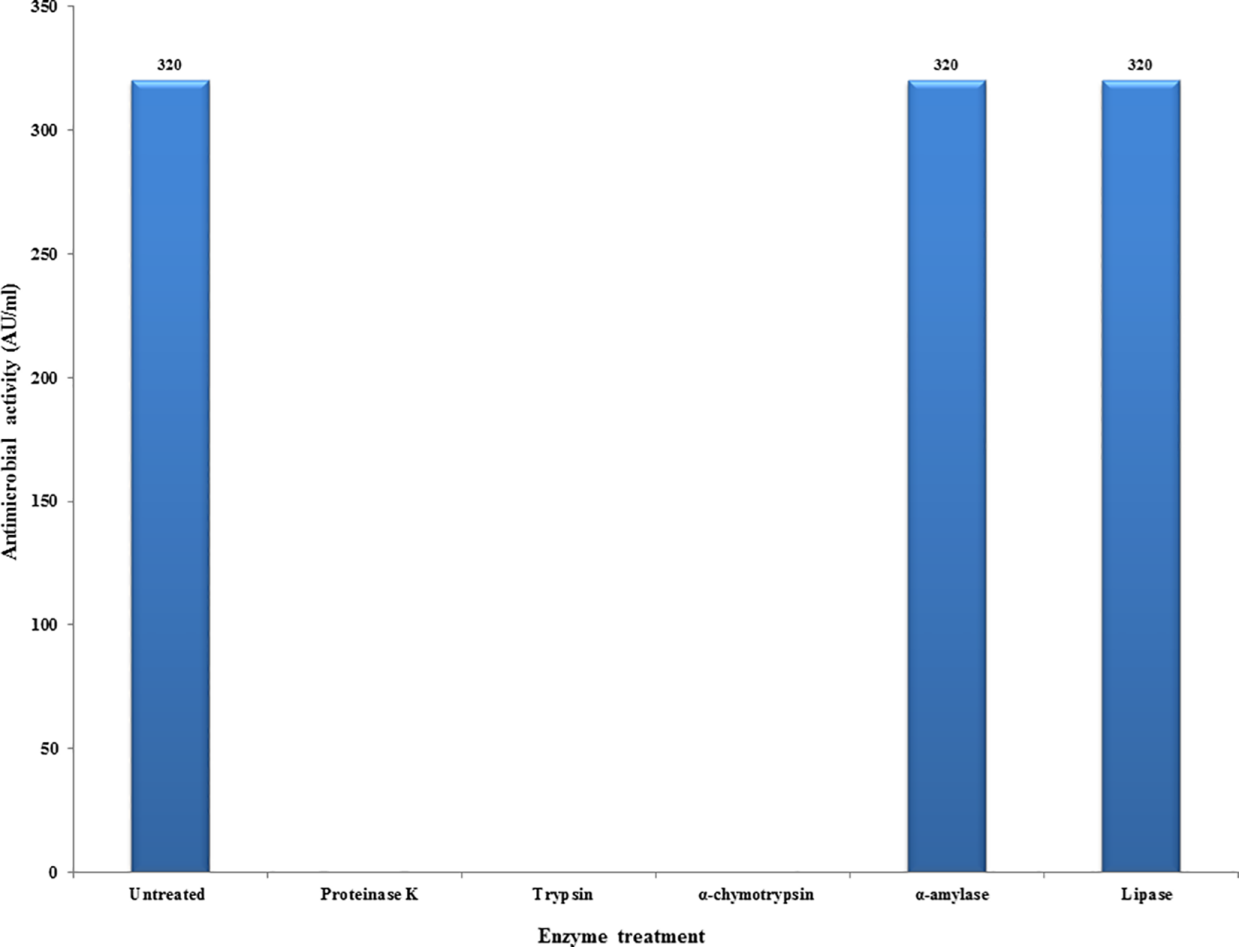
Effect of enzymatic treatments on activity of the EF478 CFS.

Protease susceptibility test was performed to confirm the nature of EF478 CFS. The antimicrobial activity of the CFS of EF478 was completely eliminated after proteinase K, trypsin and α-chymotrypsin treatment. However, its activity was preserved after incubation with amylase and lipase (Fig 1). The results indicated that the active compounds in EF478 CFS were proteinaceous in nature, and that potential carbohydrate or lipid moieties were not important for the bactericidal activity.

**Fig 2:**
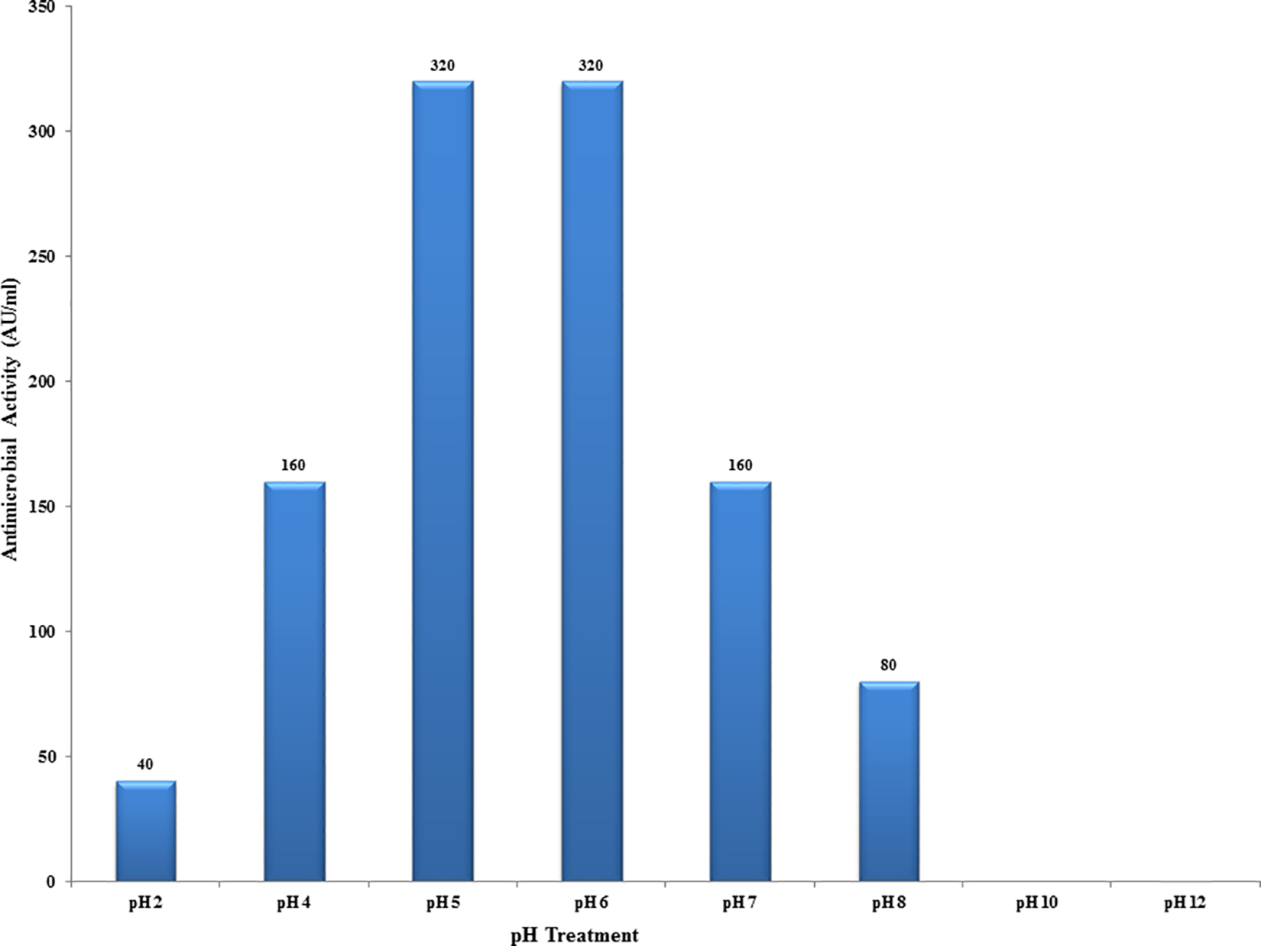
Effect of pH on antimicrobial activity of the EF478 CFS.

The bacteriocin EF478 maintained its antimicrobial activity over a 2 to 8 pH range, with a maximum of 320 AU/ml against *E*. *faecalis* ATCC 51299 at pH 5–6. At pH 4 and 7, the activity was reduced to 160 AU/ml whereas there was a significant reduction in the activity at pH 2. The activity was completely lost at pH above 10 (Fig 2).

**Fig 3:**
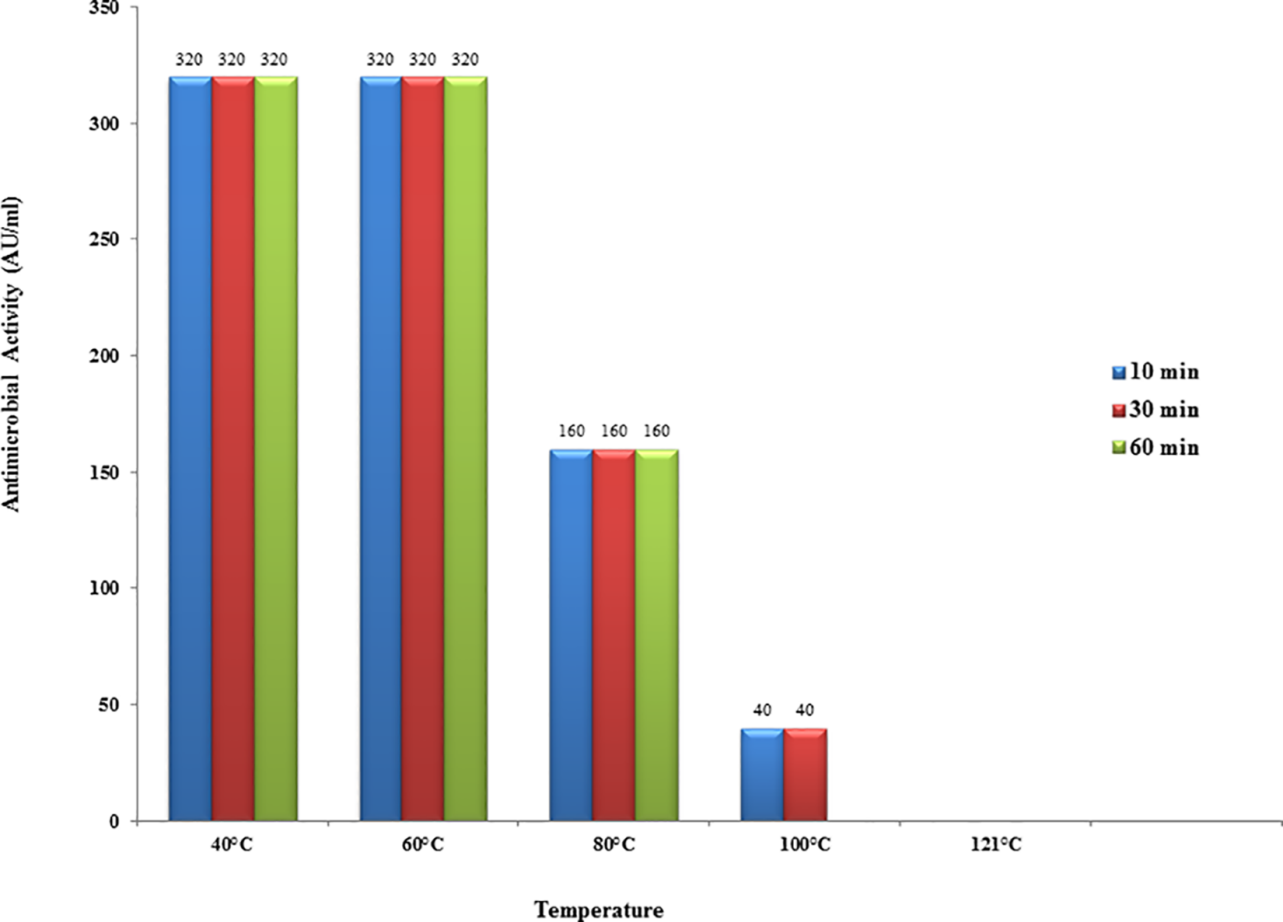
Effect of heat on antimicrobial activity of the EF478 CFS.

EF478 CFS retained its activity after 1 hour at 60°C, and was reduced to half (50%) and 12.5% after heating at 80°C for 1 hour and 100°C for 30 min, respectively. Its activity was abolished by heating at 100°C for 1 hour or following autoclaving (Fig 3).

**Fig 4:**
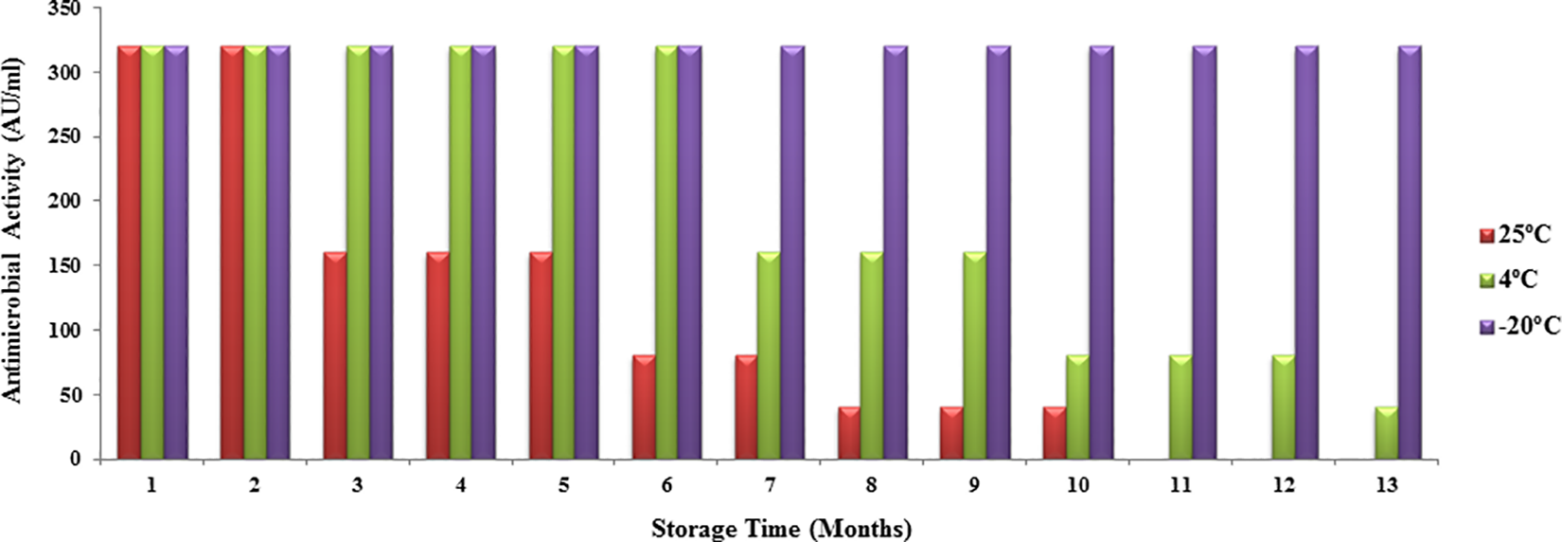
Stability after storage of the EF478 CFS.

The full antimicrobial activity of EF478 CFS was retained after storage for 1 year at -20°C, 6 months at 4°C, and 2 months at 25°C (Fig 4). The remaining activity was 50% and 25% of its initial value after storage at 4°C for 9 and 12 months, respectively. At room temperature, its activity decreased to 50% after 5 months and 25% after 7 months. The antimicrobial activity was lost after storage at 25°C for 10 months.

### Identification of the bacteriocin-producing strain

The bacteriocin-producing strain EF478 was isolated from stool samples. It was identified as *Enterococcus faecalis* by Gram staining and biochemical tests and was confirmed using the automated MicroScan system (Siemens Healthcare Diagnostics) with Lab Pro software database version 3.01 (Beckman Coulter, Sacramento, CA) and MALDI-TOF MS (Microflex, Bruker Daltonics) associated flexControl version 3.4. The highest score obtained after comparison to the predicted protein database was 2.27 and matches *Enterococcus faecalis* 104575 LDW. In addition, Kirby-Bauer disk diffusion tests showed that this strain was sensitive to antibiotics including gentamycin, teicoplanin, and vancomycin, and resistant to ampicillin, ciprofloxacin and tetracycline.

### Optimal condition for bacteriocin production by *E*. *faecalis* EF478

**Fig 5:**
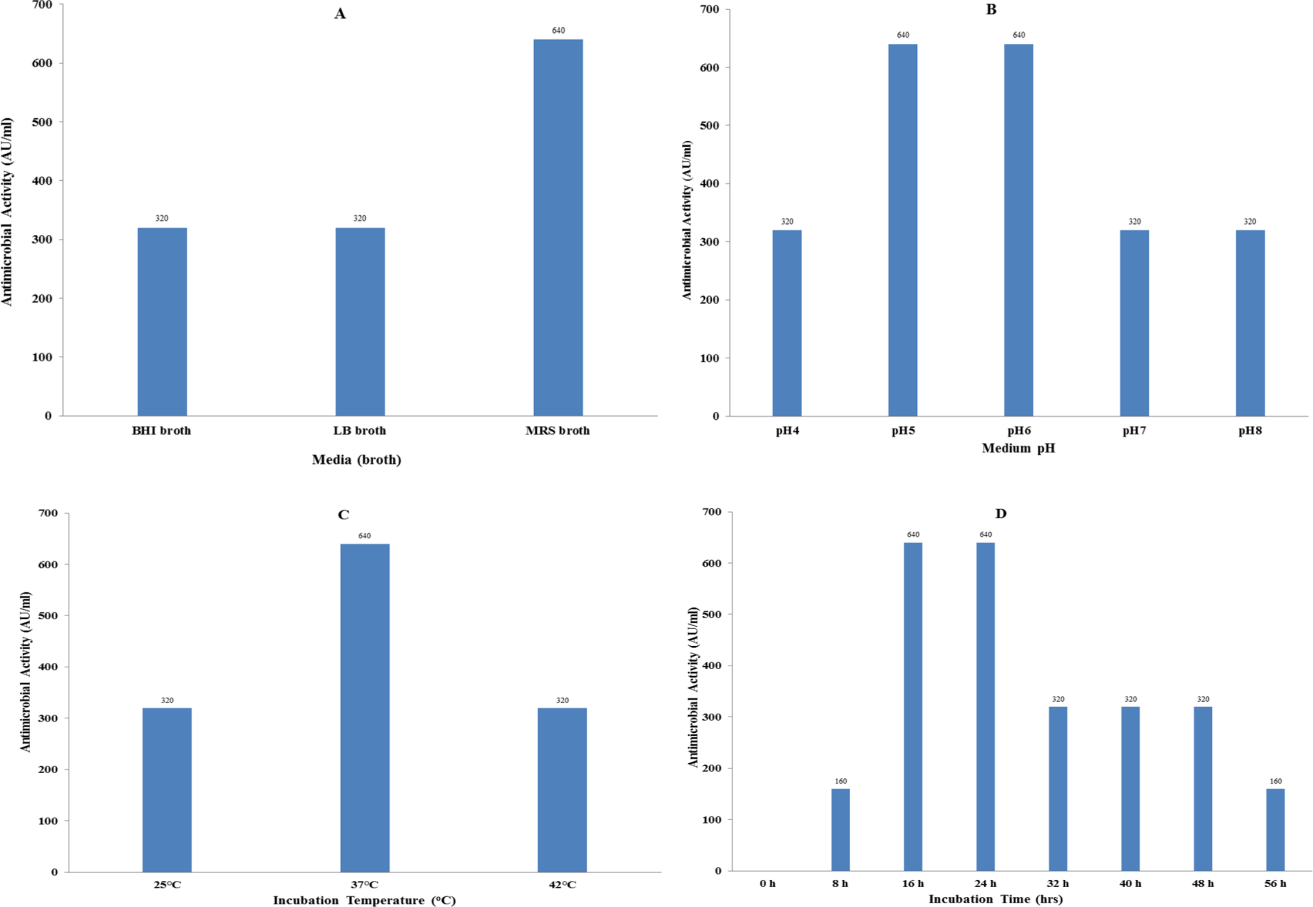
Optimization of bacteriocin-production by *E*. *faecalis* EF478. (A) Culture media (B) Medium pH (C) Incubation temperature (D) Incubation time. LB, Luria-Bertani; BHI, brain heart infusion; MRS, De Man Rogosa Sharpe. AU/ml: arbitrary unit.

To optimize bacteriocin-production and antimicrobial activity, different suitable medium and cultural conditions were tested. EF478 was grown in LB, BHI and MRS broths at 37°C for 24 hours and CFSs from each medium were tested against *E*. *faecalis* ATCC 51299. MRS medium showed the highest antimicrobial activity (640 AU/ml). Similar experiments were conducted for optimal medium pH, culture length and temperature. The highest activity (640 AU/ml) was found at pH 5 and 6, after 16–24 hours of culture at 37°C (Fig 5A–5D).

### Induction of bacteriocin EF478

Mitomycin C is commonly used to enhance the production of bacteriocins. We next measured its ability to increase bacteriocin EF478 activity using the agar well diffusion. As shown in Table 3, the antimicrobial activity of bacteriocin EF 487 doubled after addition of mitomycin C (0.25 mg/ml) in to the culture medium. No further increase was observed for mitomycin C concentration above 0.25 mg/ml, and no induction was observed below this concentration.

**Table 3:** Total proteins concentration (measured by Bradford assay) in the culture supernatants after mitomycin C treatment [27].

Mitomycin C (mg/ml)	Protein concentration (mg/ml)	Antimicrobial activity (AU/ml)
Non-induced	0.19	640
0.25 mg/ml	2.96	1,280
0.50 mg/ml	3.70	1,280
1 mg/ml	4.02	1,280

### SDS-PAGE and antimicrobial activity testing

**Fig 6:**
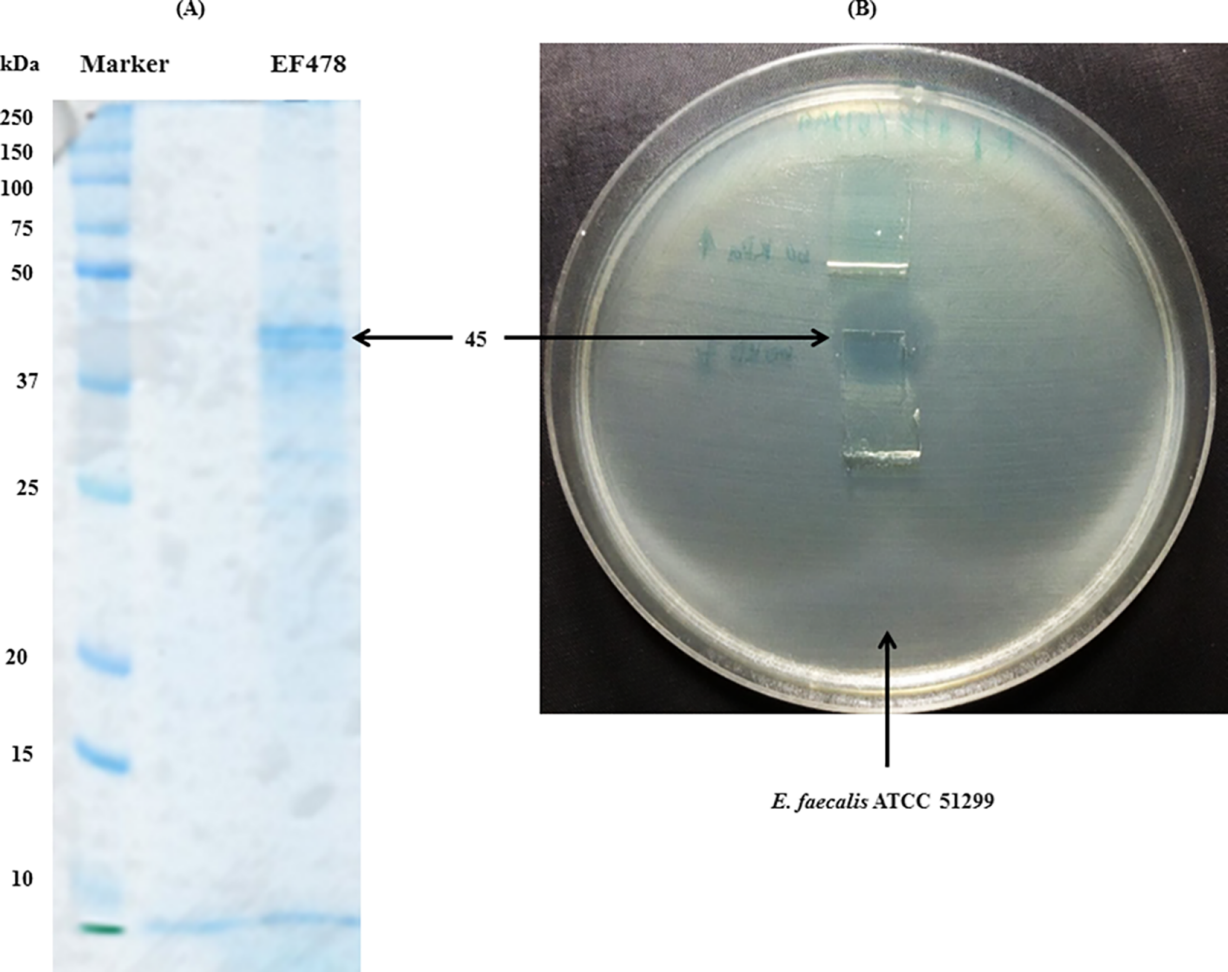
SDS-PAGE and antimicrobial activity analysis of EF478 CFS. (A): Coomassie blue staining from SDS-PAGE of the concentrated CFS of EF478. (B): Inhibition zone on the indicator lawn from the unstained gel of 45 kDa fragment.

EF478 supernatant was then fractionated and concentrated using column with different pore sizes. No antimicrobial activity was detected for the fractions with molecular weight below 10 kDa. However, potent antimicrobial activity was observed in the fraction above 10 kDa, and it corresponded with the presence of a major band of ~ 45 kDa, as determined by SDS-PAGE using protein standard (Fig 6).

### Peptide mass fingerprint identification of bacteriocin EF478

The 45 kDa protein band was cut from SDS-PAGE gel and subjected to nLC-ESI-MS/MS analysis to identify its amino acid sequence. The obtained peptide masses and fragmentation spectra were compared with the protein sequence in Genbank NCBI database using BLAST. Bacteriocin EF478 highest similarity was with a serine protease produced by *E*. *faecalis* (accession number gi|488296663, NCBI reference sequence: WP002367871.1). The amino acid sequence included 452 residues, and the calculated molecular weight of 47,809 Da corresponded to the mass (~45 KDa) measured by SDS-PAGE analysis (Fig 6). Moreover, 21 peptides were matched, 19 of which were found in the genome database of *E*. *faecalis*, covering 43% of the total sequence (S2 Fig). The sequence and some characteristics of each peptide are summarized in Table 4.

**Table 4:** Peptide fingerprinting report of EF478 using Mascot search engine.

Observed	Mr(expt)	Mr(calc)	Score	Peptide sequence
459.73	917.45	917.48	37	K.NQEISSLK.A
474.73	947.45	947.47	23	K.QSELNVMK.A
482.72	963.44	963.46	41	K.QSELNVMK.A + Oxidation (M)
600.78	1199.56	1199.59	30	R.DWEINPGITR.V
681.32	1360.63	1360.67	92	K.QLEATEAELETK.R
469.89	1406.65	1406.68	87	K.ASLALEQSSAESSK.A
704.33	1406.65	1406.68	86	K.ASLALEQSSAESSK.A
719.83	1406.65	1437.68	111	R.VGFGYSGSTIVGHSA.-
506.58	1516.73	1516.77	54	K.QLEATEAELETKR.Q
530.27	1587.79	1587.83	53	K.VKQLEATEAELETK.R
567.60	1699.80	1699.84	75	K.SEQLQQEITNLNQR.I
850.91	1699.80	1699.84	116	K.SEQLQQEITNLNQR.I
569.93	1706.77	1706.81	43	R.AAQVEAGGIPNDHWSR.G
854.39	1706.77	1706.81	23	R.AAQVEAGGIPNDHWSR.G
633.98	1898.93	1898.98	67	K.AKSEQLQQEITNLNQR.I
635.98	1904.92	1904.96	62	K.ASLALEQSSAESSKAGLEK.Q
697.33	2088.99	2089.04	32	R.VQAVSTIVSANNDLMQQQK.E+ Oxidation (M)
713.71	2138.10	2138.15	99	R.QSLGLRPVVWDAGLAASATAR.A
758.05	2271.15	2271.21	37	K.DPIPTPPPATGDVTLDALNVLR.Q
931.42	2791.25	2790.33	54	R.DVQVNGQSTTMLDAVLDADSVADAISR.V
936.75	2807.24	2806.32	68	R.DVQVNGQSTTMLDAVLDADSVADAISR.V + Oxidation (M)

Mr (expt) represented the molecular mass of peptide in experiment; Mr (calc) showed the molecular mass of peptide in calculation.

### Identification of the bacteriocin gene

**Fig 7:**
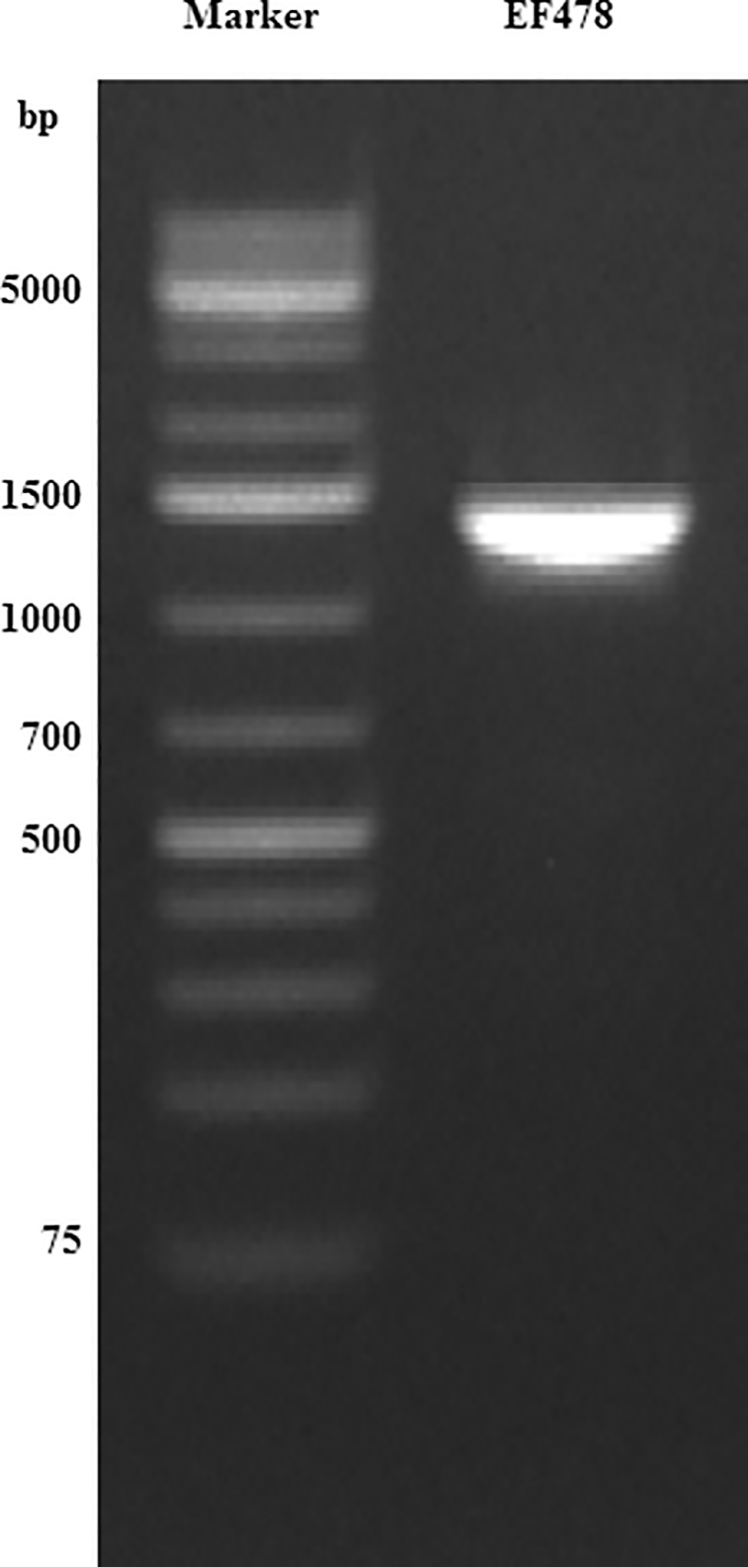
PCR amplification of *E*. *faecalis* EF478 bacteriocin gene. M: 1000-bp ladder plus, Lanes 1: PCR product of bacteriocin gene.

**Fig 8:**
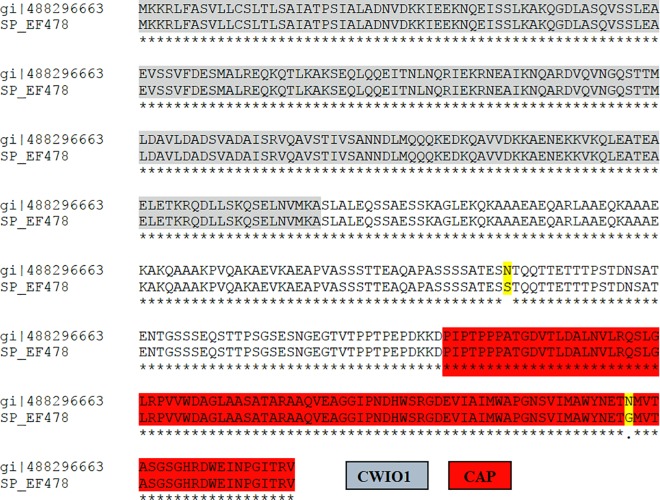
Amino acid sequence alignment of *E*. *faecalis* serine proteases WP_002367871 and the predicted EF478, using CLUSTALW. Identical amino acids are indicated by asterisks. Conserved regions of uncharacterized N-terminal domain of peptidoglycan hydrolase CwlO1 and CAP domains were highlighted with grey and red, respectively. Substituted amino acid residues were highlighted (N to S, and N to G) in yellow.

The PCR targeting the EF478 bacteriocin gene generated a single DNA fragment of expected size (1,356 bp) (Fig 7). The nucleotide sequence has been submitted to the GenBank (NCBI/Bankit/GenBank) and assigned the accession number KU641393 (S3 Fig). Its deduced amino acid was 99.53% identity with *E*. *faecalis* serine protease accession number WP_002367871 (Fig 8), which confirmed the results of the nLC-ESI-MS/MS.

### Predicted protein structure and function

**Fig 9:**
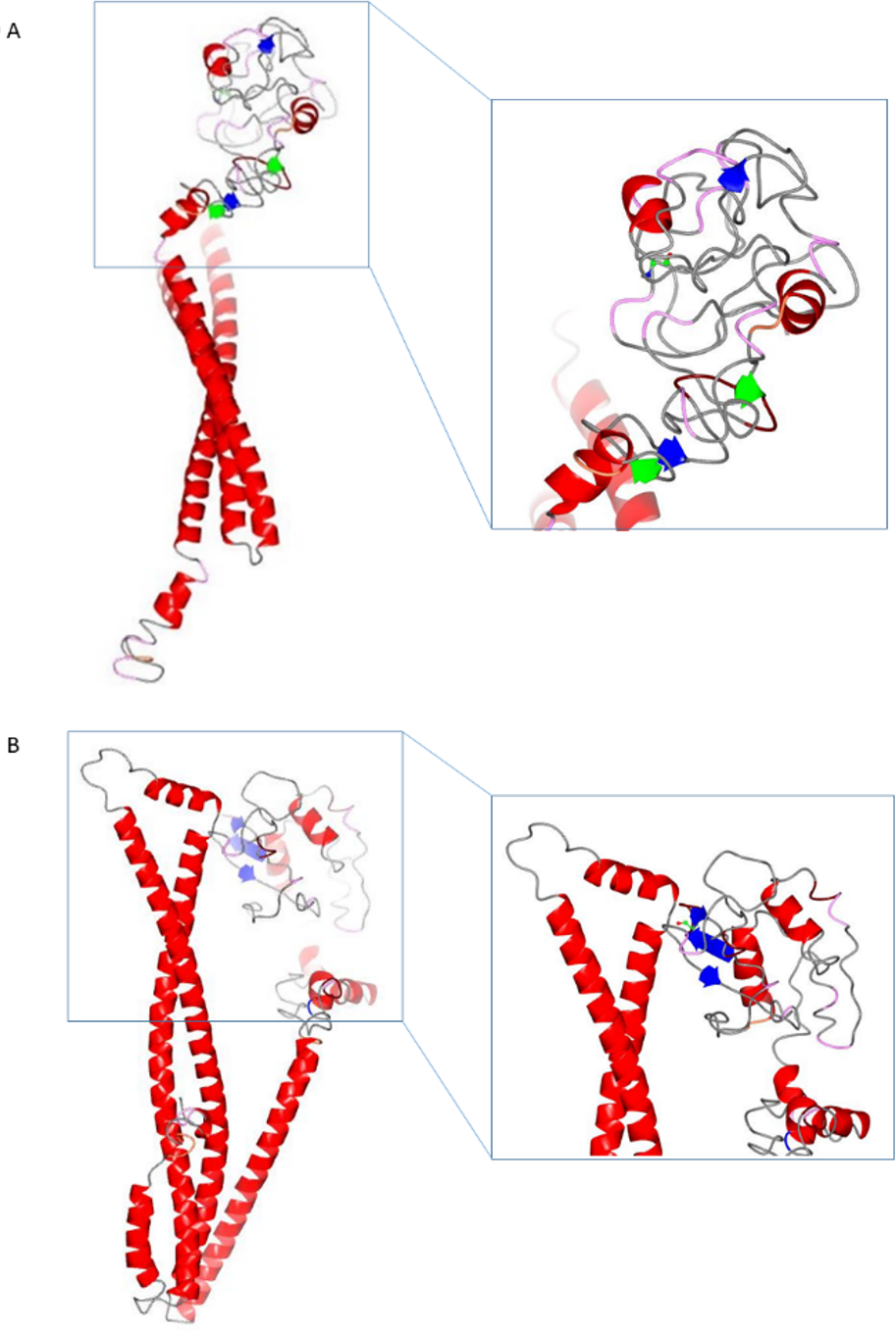
Ribbon diagrams of EF478 bacteriocin and *E*. *faecalis* serine protease. **WP_002367871:** (A) EF478 bacteriocin, (B) *E*. *faecalis* serine protease WP_002367871. The zoomed in region is the CAP superfamily domain. There is only one amino acid difference between EF478 bacteriocin and *E*. *faecalis* serine protease WP_002367871, but they have a big difference in their secondary structure and perhaps their functions. α-helices (red), β-sheets (blue) and bulge (green).

The probable protein functions of the EF478 bacteriocin was predicted by identification of conserved domains using NCBI conserved domain database (CDD) [35]. The CDD analysis revealed that the EF478 bacteriocin contains two conserved domains: a CwlO1 domain (uncharacterized N-terminal domain of peptidoglycan hydrolase CwlO) (E-value 3.82e-37) and a CAP superfamily domain (cysteine-rich secretory proteins, antigen 5, and pathogenesis-related 1 proteins) (E-value 4.02e-10) (Fig 8). The PDB files and ribbon structures of EF478 bacteriocin and the *E*. *faecalis* serine protease WP_002367871 were created and compared (Fig 9A and 9B).

## Discussion

The rapid emergence and spread of vancomycin-resistant enterococci is a threat to public health globally, as vancomycin is often the last drug of choice for severe enterococcal infections. Hence, MDRE and VRE infections are medical challenge with poor prognosis, due to the unavailability of appropriate drugs for treatment [36, 37]. In addition to antimicrobial stewardship program to solve the MDR organisms (MDRO) as well as many preventive measures, public health research focused on identifying novel antimicrobial compounds against MDROs. This study aimed to discover novel bacteriocins effective against clinical isolates of MDR- *E*. *faecium* and *E*. *faecalis*, which are the two most common causes of MDRE infections.

A total of 10,215 bacteria isolated from water, hospital laboratory environment and clinical samples were screened for their antimicrobial activity using bacterial inhibition, spot on the lawn and agar well diffusion assays. Bacteriocins commonly inhibit the growth of bacteria that are closely related to the producing strain. Therefore, we selected sampling locations where enterococci are commonly present [19, 38–42]. Bacteriocins are usually produced in response to an unfavorable environment; detection rate in the natural habitat would then be lower than in clinical settings. In this study we found 165 isolates with antimicrobial property against two indicator strains, *i*.*e*. *E*. *faecalis* ATCC 51299 (a high-level aminoglycoside and vancomycin-resistant strain) and *E*. *faecium* ATCC 35677 (a vancomycin-susceptible strain). The overall detection rate in our study (1.6%) was slightly low when compared with previous reports [19, 43, 44]. The highest proportion of antimicrobial-producing microorganisms was detected from clinical isolates (48.8%), followed by environmental isolates (10%) and water isolates (0.82%). Previous studies also reported that the highest proportion of bacteriocin-producing bacteria (40%-60%) was found in clinical samples [39, 45, 46]. The detection of bacteriocin-producers has been shown to be influenced by the method used, the sensitivity of the indicator strains to the bacteriocin, the source of bacteriocin-producer [19, 47–49]. Therefore, we used 3 different assays to detect as well as 2 different indicator strains to avoid method-based detection biased.

*E*. *faecalis* 478 isolated from stool sample showed the highest antimicrobial activity against indicators and clinical MDRE and VRE samples. Previous studies have reported that bacteriocins produced by *Enterococcus* spp. have a broad spectrum of activity to a wide range of Gram-positive bacteria [50–52]. The ability to produce bacteriocin might confer a selective advantage in bacteria communities [14, 39]. As the human gastrointestinal tract (GI) flora is one of the most dynamic and complex ecosystems, bacteriocin production could give bacteria a high competitive advantage [53]. *Enterococcus* species are lactic acid bacteria (LAB) and have been recognized as the most attractive bacteriocin-producers. They are used in food industry for food preservation and in fermentation processes [17, 52]. Culture of these bacteria is very flexible and their bacteriocins are considerably active and stable [41].

Many of bacterial interactions and the subsequent signalings occur efficiently when cells are in very close proximity [54]. Some bacteria can inhibit growth of target cells only through direct cell contact. On the other hand, bacteriocin is secreted extracellularly to kill the susceptible targeted cells, which have specific receptor for the bacteriocin. The antimicrobial activities of the CFSs from the selected isolates were determined using the commonly used methods *i*.*e*. the spot on the lawn and agar well diffusion [55].

The spot on the lawn method was a simple, rapid and easy to detect the antimicrobial activity of large number of CFS samples. The possible interfering effects of organic acids, H_2_O_2_ and bacteriophages were excluded by adjusting the supernatant to neutral pH, the addition of catalase and by performing plaque assay, respectively.

Furthermore, the agar well diffusion assay used for quantification of the bacteriocin activity also confirmed that the inhibition was not due to the presence of bacteriophages as they cannot diffuse through the agar [56].

The antimicrobial activity of CFS EF478 was inactivated by proteolytic enzymes, demonstrating its proteinaceous nature. Therefore, CFS EF478 was considered a bacteriocin and named bacteriocin EF478. Heat stability at 60°C of bacteriocin EF478 was not as high as the enterocin of *E*. *faecalis* BFE 1071 [57] (100°C for 60 min) or anti-listerial bacteriocin of *E*. *faecium* (100°C for 30 min) [58]. The pH tolerance was between 2 to 8, with a maximal antimicrobial activity at pH 5 and 6 in MRS medium. This result was comparable to some lactic acid bacteriocins presenting activities at the low pH values, of the gastrointestinal tract [59, 60]. The long-term stability of bacteriocin EF478 makes it an antimicrobial compound of interest.

Three commercial media, namely MRS, BHI and LB, were chosen to optimize EF478 production, and MRS gave the highest bacteriocin EF478. MRS, BHI and LB contain similar major ingredients for general growth requirements of enterococci including peptone, sucrose, yeast extract and sodium chloride [61–64]. The major ingredients found in MRS but absent in BHI and LB include potassium dihydrogen phosphate, magnesium sulfate, tween 80, sodium acetate and manganese sulfate [65]. Potassium dihydrogen phosphate and magnesium sulfate are pH buffering agents that control the pH of medium and provide cations used in its cell metabolism [62]. The surfactant properties of tween 80 help prevents the aggregation of bacteriocin molecules and stimulates protein secretion [66–68]. Tween 80 has also been used as an additive in the culture media of several LAB producing-strains to promote the cellular uptake of nutrients [63, 66, 67, 69]. Sodium acetate has been shown to suppress the growth of many bacteria competing with LAB. Manganese ions, a co-factor of the lactate dehydrogenase, are provided by manganese sulfate [70]. The optimal condition for bacteriocin EF478 production was observed after 16 hours of cultivation in MRS, at pH 5–6 and temperature 37°C. Bacteriocin EF478 expression was also doubled by the addition of mitomycin C (0.25–1 mg/ml).

This bacteriocin was concentrated and separated using three different sizes of ultrafiltration membrane spin-column and purified by reverse phase-chromatography. SDS-PAGE analysis revealed that a major 45 kDa protein was solely responsible for the antimicrobial activity. The band corresponding to bacteriocin EF478 was subjected to nLC-ESI-MS/MS analysis, and the identified peptides corresponded to a serine protease from *E*. *faecalis* (accession number WP_00236787). The molecular weight of the predicted protein was 47,809 Da, similar to the molecular weight observed on the SDS-PAGE gel. Moreover, a 1,356 base pair gene encoding the serine protease was amplified from the genomic DNA of *E*. *faecalis* 478. This finding strongly confirmed the presence of a serine protease produced by *E*. *faecalis* 478.

Serine proteases in prokaryotes and eukaryotes are involved in diverse processes such as digestion, blood clotting, immune responses, inflammation, cell signaling, pathogenesis, cell adhesion, metabolism, protein degradation and post-translation modification [71–78]. Several microorganisms secrete serine protease toxins, such as the subtilase-like serine protease of *E*. *coli* [76], HtrA of *Streptococcus* spp. and *E*. *coli* [60, 62, 79, 80], elastolytic of *Aspergillus fumigatus* [74], BmooSP of *Bothrops moojeni* [81], and Pro1 of *Plasmodiophora brassicae*. Therefore, the EF478 protein identified here was likely to be Bacteriocin EF478.

The CDD analysis revealed that Bacteriocin EF 478 contained two putative conserved domains: an uncharacterized N-terminal domain of peptidoglycan hydrolase CwlO (CWlO1) domain and a CAP-superfamily domain. CwlO1 are autolysins with peptidoglycan hydrolases (PHs) activity produced by bacteria. PHs usually contains a lysin motif that can interact with peptidoglycans of various Gram-positive bacteria and therefore are commonly called lysozymes [82]. CwlO plays significant role in the autolysis, cell growth, cell separation, and biofilm formation of bacteria [83, 84]. The CAP protein superfamily contains three major groups of proteins including the cysteine-rich secretory proteins (CRISPs), antigen 5 (Ag5), and pathogenesis-related 1 (PR1) and has been found in fungi, insects, reptiles, mammals and plants [85–87]. The proteins in the CAP superfamily play key roles in important biological such as the host defense, tumor suppression and fertilization [85, 88, 89].

All of the information suggested that bactericidal activity of the bacteriocin EF478 depended on the CwlO domain whereas the CAP domain played a role in protection of the producer.

The deduced amino acid of bacteriocin EF478 was nearly identity (99.53%) with *E*. *faecalis* serine protease accession number WP_002367871. There is only one amino acid difference within the CAP domain, but they have a big difference in the predicted secondary structure. As mentioned earlier that CAP domain function as immunity to the bacteriocin producer, the change within this domain might be a critical. We analyzed 25 sequences of *E*. *faecalis* serine protease from NCBI database and found that nearly all of them possessed the same amino acid with the WP_002367871. Only one strain that share the same amino acid with bacteriocin EF478 is the strain DD14 (accession number CP021161), which was reported as bacteriocinogenic lactic acid bacteria. To date, there is a scarcity of new potent antimicrobial agents for the treatment of VRE and MDRE. Serine protease from *E*. *faecalis* 478 is attractive potential therapeutic agents for the treatment of VRE and MDRE. Bacteriocin EF478 was not present in both bacteriocin databases, Bactibase and Bagel 3. Homology searches revealed that the predicted bacteriocin EF478 was novel to this species. Finally, we have discovered a novel bacteriocin from *E*. *faecalis* 478 with a potent antimicrobial activity against clinical strains of VRE. Its high activity in a wide range of pH and temperature, resilience to heat and storage stability make bacteriocin EF478 a promising candidate as an anti-MDRE and VRE antimicrobial.

## Supporting information

S1 Fig Flow chart of the study. (DOCX)

S2 Fig Predicted amino acid sequences of serine protease of *E*. *faecalis* (NCBI accession number gi|488296663). The amino acids in bold red indicated the matched peptides obtained by the MASCOT database search with the ESI tandem MS data.
(DOCX)

S3 Fig The nucleotide sequence of serine proteases of *E*. *faecalis* 478 (Accession number KU641393). (DOCX)

S1 Table List of sampling locations, geographic coordinates and sample sizes. (DOCX)
