# Supplementary material for: A novel bacteriocin from Enterococcus faecalis 478 exhibits a potent activity against vancomycin-resistant enterococci
Source: PLoS One. 2017 Oct 12;12(10):e0186415. doi: 10.1371/journal.pone.0186415 (PMC5638566; doi:10.1371/journal.pone.0186415)
Supplement: S1 Fig — (DOCX) [file pone.0186415.s001.docx]

**Supporting Information**

Collection of samples

(Water, lab bench surfaces, and stool)

Screening for the bacteriocin producing bacteria by bacterial inhibition method

Selection of the potential isolates to prepare CFSs

Screening the antimicrobial activity of CFSs by spot on the lawn

Selection of the potential CFSs to confirm the activity by agar well-diffusion method

Determination of CFSs activity against MDRE and VRE isolates

Selection of the highest potential CFS (EF 478)

Identification of bacteriocin-producing strain

Characterization of the bacteriocin

Purification of the bacteriocin production

Optimization of bacteriocin production

Identification of protein and the gene coding bacteriocin

**S1 Fig.** Flow chart of the study
