# Supplementary material for: A novel bacteriocin from Enterococcus faecalis 478 exhibits a potent activity against vancomycin-resistant enterococci
Source: PLoS One. 2017 Oct 12;12(10):e0186415. doi: 10.1371/journal.pone.0186415 (PMC5638566; doi:10.1371/journal.pone.0186415)
Supplement: S2 Fig — The amino acids in bold red indicated the matched peptides obtained by the MASCOT database search with the ESI tandem MS data. (DOCX) [file pone.0186415.s002.docx]

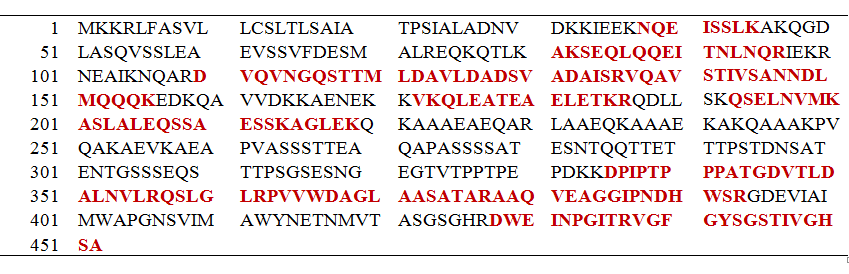


**S2 Fig.** Predicted amino acid sequences of serine protease of *E. faecalis* (NCBI accession number gi|488296663). The amino acids in bold red indicated the matched peptides obtained by the MASCOT database search with the ESI tandem MS data.
