# Supplementary figures and images for: A novel bacteriocin from Enterococcus faecalis 478 exhibits a potent activity against vancomycin-resistant enterococci
Source: PLoS One. 2017 Oct 12;12(10):e0186415. doi: 10.1371/journal.pone.0186415 (PMC5638566; doi:10.1371/journal.pone.0186415)

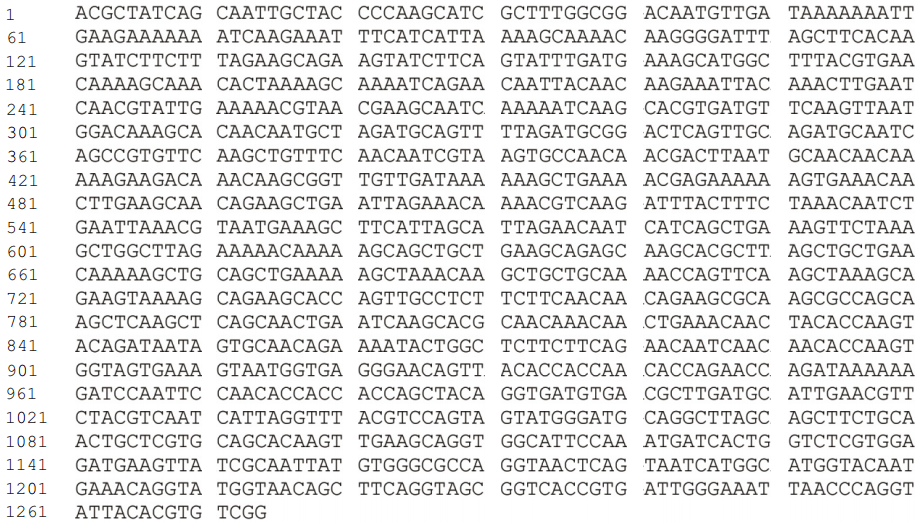


**S3 Fig** The nucleotide sequence of serine proteases of *E. faecalis* 478

(Accession number KU641393).

Supplement: S3 Fig — (DOCX) [file pone.0186415.s003.docx]
