# Supplementary material for: A novel bacteriocin from Enterococcus faecalis 478 exhibits a potent activity against vancomycin-resistant enterococci
Source: PLoS One. 2017 Oct 12;12(10):e0186415. doi: 10.1371/journal.pone.0186415 (PMC5638566; doi:10.1371/journal.pone.0186415)
Supplement: S1 Table — (DOCX) [file pone.0186415.s004.docx]

**S1Table.** List of sampling locations, geographic coordinates and sample sizes.

|  | Sampling location | Country | Long E | Lat N | Sample size |
| --- | --- | --- | --- | --- | --- |
| 1 | Mahasawat Canal | Thailand | 100.237 | 13.990 | 2,790 |
| 2 | Bangtal Canal | Thailand | 100.419 | 13.793 | 2,385 |
| 3 | Chao Phraya River | Thailand | 100.503 | 13.783 | 2,295 |
| 4 | Pasak River | Thailand | 100.580 | 14.314 | 2,430 |
